# Coffee intake and risk of diabetic nephropathy: a Mendelian randomization study

**DOI:** 10.3389/fendo.2023.1169933

**Published:** 2023-07-04

**Authors:** Jiaxi Fang, Kai Song, Di Zhang, Yan Liang, Huan Zhao, Juan Jin, Qiang He

**Affiliations:** ^1^Zhejiang Provincial People’s Hospital, Qingdao University, Hangzhou, Zhejiang, China; ^2^Nephrology Center, Department of Nephrology, Zhejiang Provincial People’s Hospital, Affiliated People’s Hospital, Hangzhou Medical College, Hangzhou, Zhejiang, China; ^3^Geriatric Medicine Center, Department of Endocrinology, Zhejiang Provincial People’s Hospital, Affiliated People’s Hospital, Hangzhou Medical College, Hangzhou, Zhejiang, China; ^4^Department of Nephrology, The First Affiliated Hospital of Zhejiang Chinese Medical University, Zhejiang Provincial Hospital of Traditional Chinese Medicine, Hangzhou, Zhejiang, China

**Keywords:** coffee intake, diabetic nephropathy, Mendelian randomization, causality, risk

## Abstract

**Rationale and objective:**

A causal relationship concerning coffee intake and diabetic nephropathy (DN) is controversial. We conducted a Mendelian randomization study to assess the causal nature of these associations.

**Methods:**

40 independent single nucleotide polymorphisms (SNPs) associated with coffee intake were selected from the UK Biobank study. Summary-level data for diabetic nephropathy were obtained from publicly available genome-wide association studies (GWAS) and the FinnGen consortium. Inverse variance weighted (IVW), MR-Egger, and weighted median (WM) methods were used to examine a causal association. Sensitivity analyses included Cochran’s Q test, the intercept of MR-Egger, MR-PRESSO, and the Outlier method. Leave-One-Out sensitivity analyses were also conducted to reduce the heterogeneity.

**Results:**

Our current study demonstrated positive associations of genetically predicted coffee intake with diabetic nephropathy (OR=1.939; *P* = 0.045 and type 2 diabetes with renal complications (OR = 2.787, *P*= 0.047). These findings were robust across several sensitivity analyses.

**Conclusions:**

This study found a positive correlation between coffee consumption and the risk of diabetic nephropathy using genetic data. For a more accurate and trustworthy conclusion, subgroup analysis on coffee intake, including preparing method, variety of coffee, and quantity, is required.

## Introduction

Diabetes mellitus (diabetes) is a growing public health concern associated with significant health care expenses and mortality. Epidemiological studies claim that diabetes affects more than 425 million individuals worldwide, and its prevalence and incidence are growing(1), thus suggesting the concept of a diabetic pandemic. Diabetic kidney disease, also known as diabetic nephropathy, is a prevalent and refractory chronic microvascular complication in diabetic patients, and the primary cause of end-stage renal disease (ESRD) worldwide(2). The World Health Organization (WHO) estimates that the number of diabetic patients will increase to 700 million, and more than 1/3 of diabetic patients with clinically develop DKD patients until 2045, a significant cause of disability and death in diabetic patients(3). Early DKD is characterised by glomerular membrane dilatation, increased glomerular filtration rate, microalbuminuria, podocyte loss, increased basement membrane thickness, glomerular and renal tubular cell damage, which leads to glomerular sclerosis and interstitial fibrosis, and ultimately renal failure. (4). Therefore, strategies to prevent and treat diabetic nephropathy are urgently needed. The number of diabetics with end-stage renal disease continues to rise despite intensive efforts to find pharmacological therapies to halt the progression of the disease (5). However, many medications have adverse effects; therefore, dietetic therapy or a highly effective and low-toxic medication is a novel approach to the treatment of diabetic nephropathy.

Coffee is the world’s most beloved drink, second only to water, with an estimated consumption value of 10 billion US dollars globally. Coffee is a commonly ingested beverage consisting of a complex mixture of compounds, such as caffeine, chlorogenic acid, and diterpenes (6). Drinking coffee can be both beneficial and detrimental to body health. The health benefits of coffee are predominantly attributable to its high plant ingredient content, including caffeine and chlorogenic acid (7, 8). Caffeine is one of the major pharmacologically active compounds found in coffee, which has both positive and negative effects on human health. The equilibrium between caffeine’s positive and negative health effects depends largely on an individual’s susceptibility to its effects(6).

Although there is still no solid evidence linking coffee intake to a higher risk of heart disease and hypertension (9, 10), coffee consumption is inversely linked to dementia, developing insulin resistance, type 2 diabetes, cirrhosis, and an increased risk of osteoporosis (11–15). Coffee may affect the risk of chronic diseases such as melancholy, type 2 diabetes, and Parkinson’s disease, according to the majority of current research (16, 17). Epidemiological and observational research have explored the relationship between coffee consumption and the risk of T2DM and discovered that persons who consume more coffee had greater glucose tolerance and a much lower risk of T2DM(18). Although the evidence is inconclusive, a higher coffee intake has been associated with enhanced kidney function. Observational studies have found either an association between higher coffee consumption and a lower risk of chronic kidney disease (CKD) (19, 20), albuminuria (21), or kidney failure (22)or no association with CKD(23). However, other studies have found that coffee consumption can impair glucose tolerance and reduce insulin sensitivity(24). There is evidence that caffeine consumption (5 mg/kg BW) causes diminished insulin sensitivity and acute insulin-insensitive environments in type 2 diabetics, thus disrupting blood glucose homeostasis in response to high and low glycaemic index meals in healthy men(25). In addition, caffeine in coffee may interact with adenosine receptors, interfering with adenosine’s anti-inflammatory and glomerular hemodynamic effects, leading to albuminuria and glomerular remodelling and sclerosis(26, 27). To summarize, their results were not consistent. In addition, most studies have focused on the relationship between coffee intake, blood glucose level, diabetes risk, and diabetes-related complications, especially diabetic nephropathy, which were rarely included as outcome indicators.

Mendelian randomization (MR) is a research technique employed to study causal relationships. It is based on the principle of genetics, where the inheritance of genes is random. The technique employs genetic variants that are related to a risk factor to validate the assumption that this risk factor is responsible for a particular outcome. Because genetic variants are arbitrarily inherited, they can be used as a surrogate for exposure to the risk factor, providing a natural experiment for testing causality(28). Since it reduces the likelihood of confounding, reverse causality, and bias due to measurement error, it can effectively avoid confounding and reverse causal bias discrimination and provide more substantial evidence of causality to traditional epidemiological studies(29). Overall, MR is a valuable tool for causal inference and provides a complementary approach to formal observational studies, especially when conventional confounding control methods are insufficient.

Consequently, the purpose of this study was to investigate the genetic causality between coffee consumption and diabetic nephropathy using a two-sample MR analysis based on the GWAS database.

## Materials and methods

### Study design

GWAS data on coffee intake and four outcomes related to diabetic nephropathy were acquired by the publicly available GWAS catalog, UK Biobank (www.nealelab.is/uk-biobank), FinnGen database (www.finngen.fi/fi) and CKDgen consortium.Because of the re-analysis of previously summarized data, no additional ethical approval was required. We used R (version 4.2.1) and Two-sample MR (version 0.5.5) to conduct a two-sample MR analysis.

**Figure 1:**
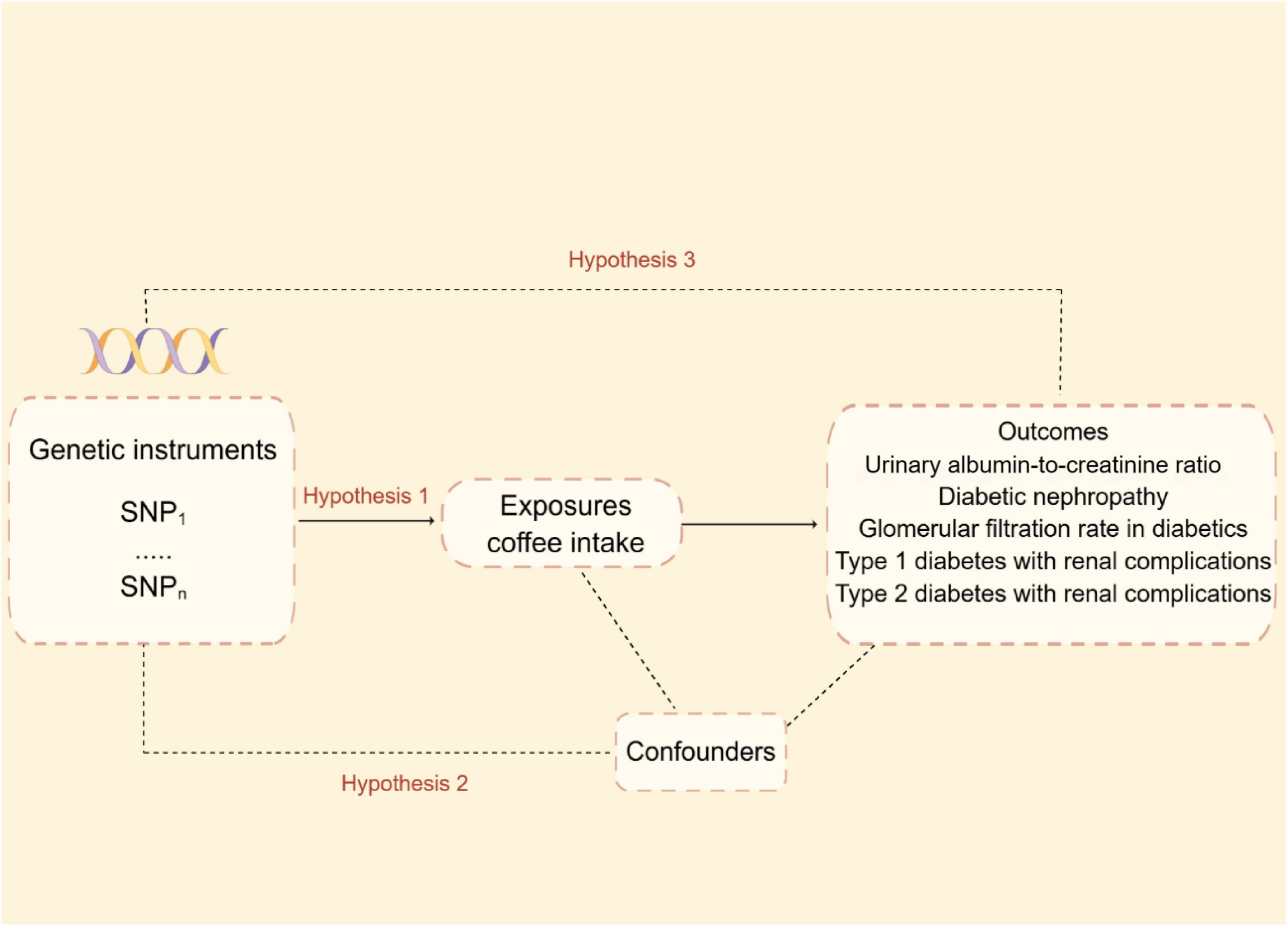
Mendelian randomization model and three fundamental assumptions of a Mendelian randomization analysis. SNP, single nucleotide polymorphisms.

Mendelian randomization relies on three fundamental hypotheses:1) The Instrumental Variable Hypothesis: The genetic variant selected as the instrumental variable (IV) is unintentionally related to the exposure of interest in a casual manner. 2) The used genetic variants should not be associated with potential confounding variables in the exposure–outcome relationship. 3) The Pleiotropy Hypothesis: The genetic variant used as the IV is associated solely with the outcome *via* its effect on the exposure and no other biological pathways. **Figure 1** depicts the MR design’s flowchart.

To develop genetic instruments for coffee consumption and diabetic nephropathy, we identified SNPs (single nucleotide polymorphisms) that are reliable (*P*< 5 × 10^−8^) and independent (r^2^< 0.001, kb = 10,000) of coffee intake. PhenoScanner V2 (www.phenoscanner.medschl.cam.ac.uk) was used to remove the SNPs related to potential confounders of Diabetic nephropathy. We compute the F statistic to determine the reliability of each genetic instrument. If the F-statistic is greater than 10, the association between IV and exposure was strong. The F-statistic for each SNP was calculated as follows:


$$F=\left(\frac{R^{2}}{1-R^{2}}\right)*\left(\frac{n-k-1}{k}\right)$$

R^2^ was calculated as follows:


$$R^{2}=2*\left(1-MAF\right)*MAF*{\left(\beta \right)}^{2}$$

R^2^: the cumulative explained variance of the selected IVs on exposure; EAF: the effect of allele frequency

β: estimated effect of SNP; N: sample size of the GWAS

### Genetic instrument selection

From genome-wide association studies (GWAS) on coffee consumption in the GWAS database with up to 428,860 individuals of European ancestry, 40 single-nucleotide polymorphisms (SNPs) were associated with coffee consumption. To avoid potential bias due to substantial linkage disequilibrium (LD), we chose SNPs with LD r^2^< 0.001. F statistics were calculated to ensure the strong correlation between SNP and exposure, and SNPs with F > 10 were extracted.

### Data source for diabetic nephropathy

The detailed information of genome-wide association studies included in this paper were listed in **Table 1**. Diabetic nephropathy was defined as an outcome when glomerular disorders in patients with diabetes mellitus met the ICD-10 (code: N08.3*) criterion, with summary statistics from the FinnGen biobank including 213,746 European individuals (3,283 cases and 2,10463 controls). We also collected the aggregated data on glomerular filtration rate in diabetics from a previous study containing 144,935 participants of European (30). We extracted summary statistics for type 2 diabetes with renal complications from the FinnGen database, which included 1,296 cases and 183,185 European-descent controls. The GWAS data for type 1 diabetes with renal complications were derived from another summary-level GWAS data in FinnGen with a total of 963 cases and 183,185 controls. The GWAS summary data for Urinary albumin-to-creatinine ratio were extracted from the CKDgen consortium (http://ckdgen.imbi.uni-freiburg.de/), which included 5,825 cases and 46061 controls of European individuals.

**Table 1:** Details of the GWAS studies included in the Mendelian randomization.

Year	Trait	Population	Sample size	Web source
2018	Coffee intake	European	428,860	www.nealelab.is/uk-biobank
2021	Diabetic nephropathy	European	184,987	www.finngen.fi/fi
2016	Glomerular filtration rate in diabetics	European	144,935	DOI: 10.1038/ncomms10023 PMID: 26831199
2021	Type2 diabetes with renal complications	European	184,481	www.finngen.fi/fi
2021	Type 1 diabetes with renal complications	European	184,148	www.finngen.fi/fi
2015	Urinary albumin-to-creatinine ratio in diabetes	European	51,886	http://ckdgen.imbi.uni-freiburg.de/ PMID: 26631737

### Statistical analysis

The inverse variance weighting (IVW), MR-Egger, and weighted median (WM) methods were used to examine a causal association, with IVW being the primary analytical method (31). IVW method can achieve unbiased causal estimates without horizontal pleiotropy where the variants affect the direction and outcome through pathways that are not on the causal pathway of interest (32). Therefore, the results of the IVW method are the most accurate. The weight median method is less sensitive to outliers and measurement errors than other methods. The MR-Egger method regresses the genetic variant-outcome associations on the genetic variant-exposure associations, allowing for the presence of pleiotropy, so it can provide a valid causal estimate even in the presence of pleiotropy, as long as certain assumptions are met. In conclusion, the WM and MR-Egger methods were performed as additional tests for the MR estimates.

Several sensitivity analyses were conducted to obtain stable MR estimates. The IVW and MR-Egger were utilised to quantify the heterogeneity effect among genetic instruments (33). The Cochran’s Q test was used to evaluate the heterogeneity of a subset of genetic IVs. The vertical pleiotropy was evaluated by the intercept obtained by MR-Egger regression (34). MR-Pleiotropy Residual Sum and Outlier method (MR-PRESSO) was also performed to detect the outliers and potential horizontal pleiotropy (global *P*< 0.05 shows the presence of horizontal pleiotropy). Finally, the leave-one-out method was utilised to address sensitivity analysis.

**Figure 2:**
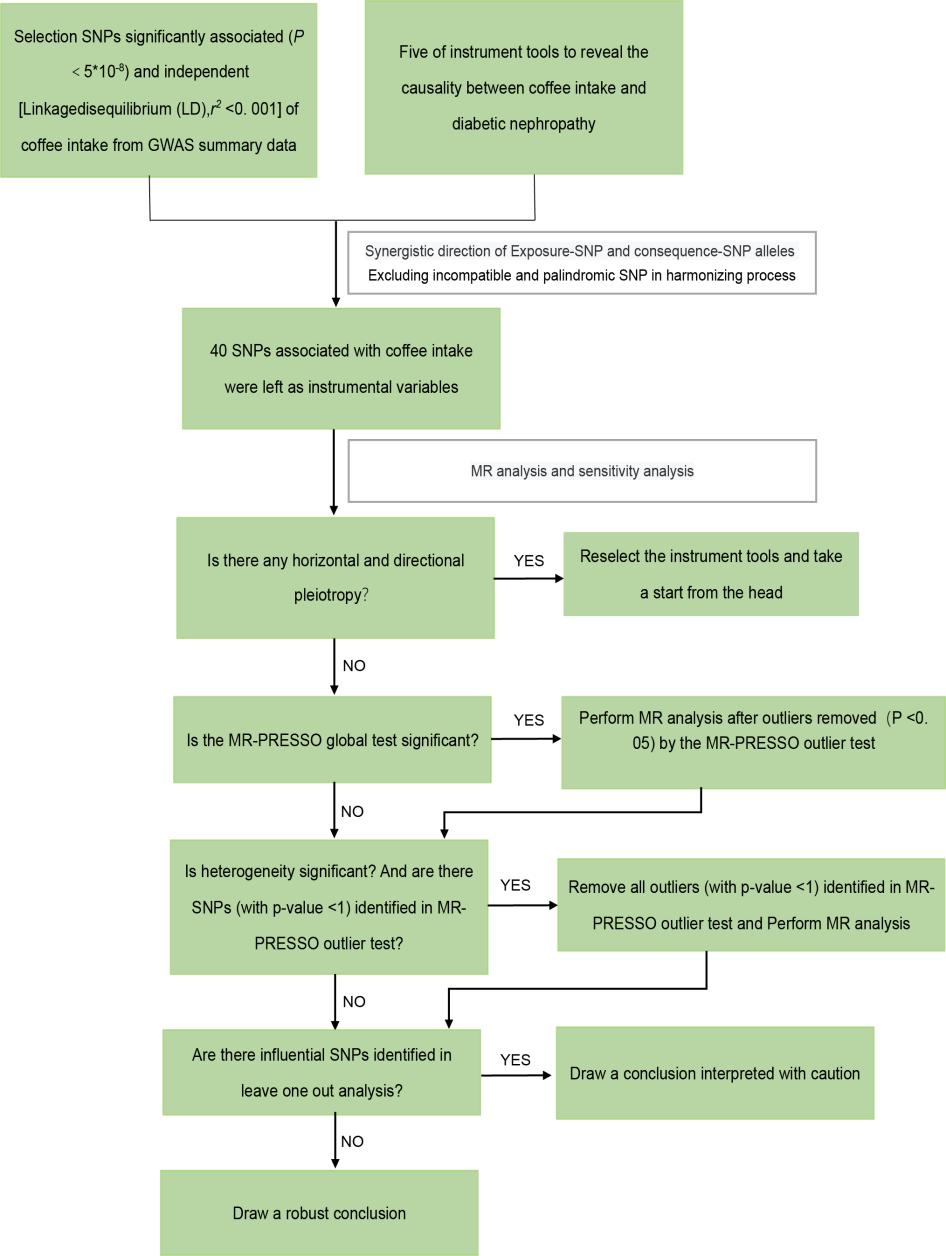
The framework of the Mendelian randomization analysis. SNP, single nucleotide polymorphisms.

According to the guidelines stated by STROBE-MR (35), we performed a two-sample MR study. The overall workflow of our two-sample MR study is set out in **Figure 2**.

## Results and discussion

### GWAS data on coffee intake

Summary-level GWAS data with coffee intake was obtained from the UK-biobank (www.nealelab.is/uk-biobank). As genetic instrumental variables, we extracted 38 SNPs (single nucleotide polymorphisms) that are reliable (*P*< 5 × 10^−8^) and independent (r^2^< 0.001, kb = 10,000) of coffee consumption eventually. Two SNPs (rs1421085 and rs476828) were disregarded by PhenoScanner V2 because they were associated with known confounding factors (diabetes and HbA1c). These SNPs and the strength and magnitude of their associations with coffee intake are shown in **Table S1**. There was no correlation between outcome variables and instrument variables, indicating the absence of instrument bias. The heterogeneity test stated the existence of heterogeneity in diabetic nephropathy and type 2 diabetes with renal complications, so we removed all outliers (with p-value<1) identified in the MR-PRESSO outlier test and reperform MR analysis. After coordinating the allelic directions of exposure-SNP and outcome-SNP and eliminating palindromic SNP and incompatible SNP according to the size of EAF, we finally got a summary table of SNPs for coffee intake and diabetic nephropathy. Refinement statistics are shown in **Tables S2.1**- **S2.5**.

### The results of the two-sample MR analysis

**Figure 3:**
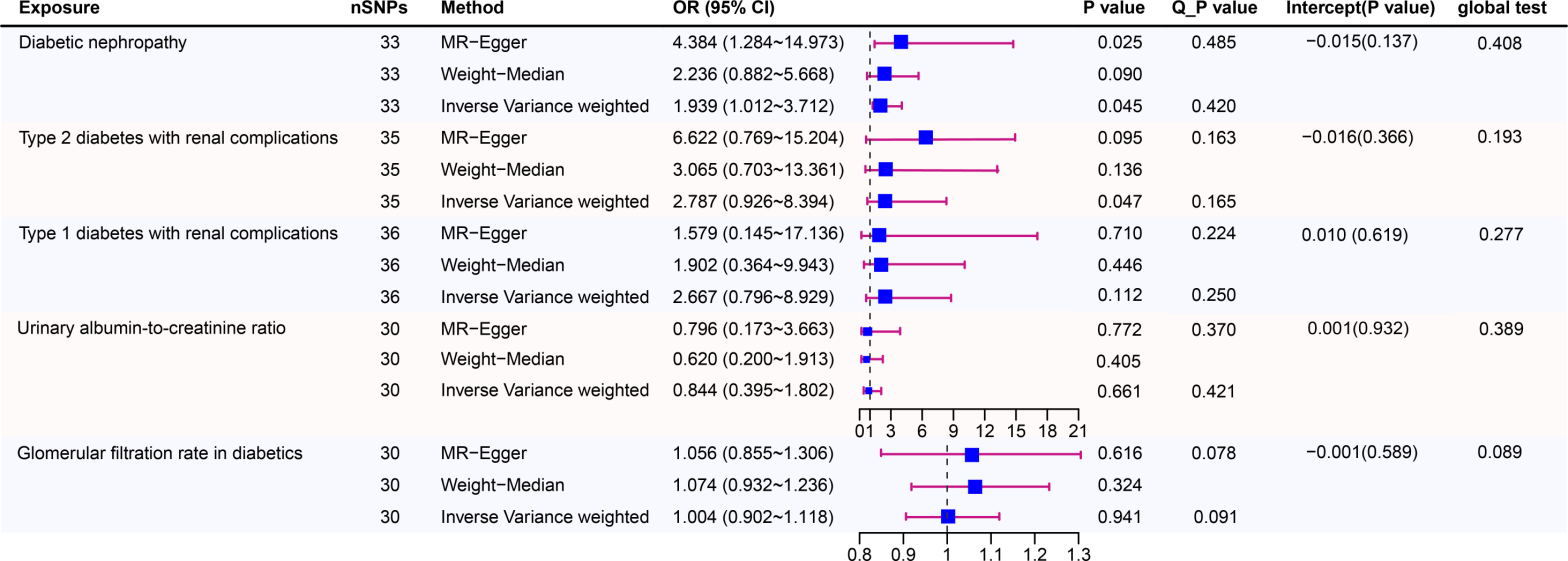
Causal risk between coffee intake and Diabetic Nephropathy was estimated using conventional inverse variance weighted (IVW) Mendelian randomization analysis, Egger-Mendelian randomization (MR-Egger), and weighted median Mendelian randomization. Forest plot and sensitivity analysis of Mendelian randomization analyses showing the effect of coffee intake on the risk of diabetic nephropathy.

**Figure 4:**
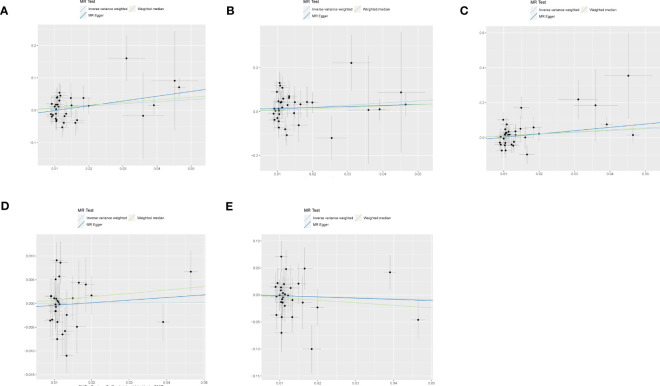
The scatter plot for MR analyses of causal associations between each coffee intake SNP and Diabetic nephropathy **(A)**, type 1 diabetes with renal complications **(B)**, type2 diabetes with renal complications **(C)**, glomerular filtration rate in diabetics **(D)**, urinary albumin-to-creatinine ratio **(E)**.

**Figure 3** shows estimates of the causal effects of coffee intake on diabetic nephropathy and forest plots of the estimates for each outcome using the different MR methods. Associations for individual SNPs in detail are presented in Supplementary **Table S2**. The causal effect estimate for each SNP on diabetic nephropathy is visualised using scatter plots of the SNP-outcome relationships and the SNP-coffee associations in **Figure 4**.

In a study of coffee intake and diabetic nephropathy, the odds ratio (OR) of IVW analysis was 1.939 (95% confidence interval [CI], 1.012-3.712; *P* = 0.045). The result of MR-Egger (OR= 4.384, *P*= 0.025) was consistent with IVW, indicating a significant positive causal connection were identified in coffee intake and diabetic nephropathy. When assessing heterogeneity, no indication of heterogeneity was found (Cochran’s Q *P*= 0.420). The results of MR-Egger and MR-PRESSO did not suggest any evidence of horizontal pleiotropy (*P* for MR-Egger regression intercept=0.137 and global test *P*= 0.408). The finding was robust in the leave-one-out sensitivity analysis (**Figure S2A**).

To provide a more nuanced study of coffee’s casual effects on diabetic nephropathy, we analysed the two types of diabetic renal complications. For type 2 diabetes with renal complications, a weak significant causality was observed. IVW showed that the effect of coffee intake on type 2 diabetes with renal complications was statistically significant (OR = 2.787, 95% CI: 0.926-8.394; *P* = 0.047), the MR-Egger (OR=6.622, *P*=0.095) and WM analysis method results (OR= 3.065, *P*=0.136) were inconsistent with IVW method however. Heterogeneity tests showed that there was no heterogeneity (Cochran’s Q *P*= 0.165). And no evidence for directional horizontal pleiotropy was observed (*P* for intercept=0.366, global test *P*=0.193). The robustness of the outcomes was determined using the leave-one-out approach (**Figure S1C**). For type 1 diabetes with renal complications, the situation is dissimilar. A causal relationship was not found between coffee intake and type 1 diabetic renal complications (IVW: OR = 2.667, 95% CI: 0.796-8.929, *P*= 0.112). Likewise, there was no clear evidence of heterogeneity (Cochran’s Q *P*= 0.250) or pleiotropy (Egger intercept = 0.010, *P* = 0.619; global test *P*= 0.277). The leave-one-out sensitivity analysis validated the stability of the results (**Figure S2B**).

As for the glomerular filtration rate in diabetics, no clear causal relationship was found. Random effects IVW estimates indicated that a high level of coffee intake is not causally associated with a higher risk of glomerular filtration rate in diabetics (OR=1.004, 95% CI: 0.902-1.118, *P*=0.941). Similar to the results of IVW analyses, the MR-Egger and WM analyses suggested a relationship of a non-distinctive character between coffee intake and glomerular filtration rate in diabetics. The *P* value of Cochran’s Q test was 0.091, furthermore the results of the pleiotropy study performed using the MR-Egger intercept test and MR-PRESSO revealed that none of the 30 genetic variations exhibited any discernible pleiotropy (Egger intercept = -0.001, *P* = 0.589; global test *P*= 0.089). Similar to the glomerular filtration rate, urinary albumin-to-creatinine ratio also did not find a significant causal association (OR=0.884, 95% CI: 0.395-1.802, *P*=0.661). In addition, the MR-Egger (OR =0.796, 95% CI = 0.173–3.663, *P* = 0.772) and WM (OR = 0.620,95% CI = 0.200–1.913, *P* = 0.405) methods reached the same conclusion. Cochran’s Q test and MR-Egger regression indicates there is no significant heterogeneity or vertical pleiotropy in our MR analysis. (Cochran’s Q *P* =0.421, Egger intercept =0.001, *P* = 0.932; global test *P*= 0.389)

## Discussion

The current study examined the relationship between coffee consumption and diabetic nephropathy using two sample MR analyses. Our current study demonstrated positive associations of genetically predicted coffee intake with the risk of diabetic nephropathy in 428,860 participants of European ancestry.

Our finding on coffee intake concerning diabetic nephropathy aligns with some but not all previous studies. The study by Xiu et al. consisted of 7 randomised controlled trials (RCTs) in which caffeine intake was the sole variable. The researchers discovered that acute caffeine ingestion significantly decreased the insulin sensitivity index, with a standardised mean difference of 2.06 (95% confidence interval 2.67 to 1.44, I2 = 49%, P for heterogeneity = 0.06) (36). The short-term trials also demonstrated a transient impairment in the 2–3h postprandial glucose response after coffee consumption. In comparison to water, caffeinated beverages may have a short-term effect on the glycemic AUC response (37). Coincidentally, when we investigated the association by coffee subtypes, individuals who consumed > 2 cups/day of caffeinated coffee revealed a more significant eGFR decline and a higher risk of accelerated kidney function decline than those who consumed none to 1 cup/day (38). Renata et al. also demonstrated that high caffeine intake exacerbates renal fibrosis and apoptosis in a mouse model that is homologous to human disease (Pkd1cond/cond:Nestincre) (39). A cohort study that included 407 participants with AER records both at baseline and end of follow-up found that the risk of developing albuminuria (AER >30 mg/24 h) in slow metabolizers increased significantly for heavy coffee drinkers(40). A systematic review of two subsamples of atherosclerosis risk in communities detected coffee-associated metabolites positively associated with coffee consumption and higher risk for incident CKD (41). In a meta-analysis of two subsamples from an atherosclerosis risk study, William J et al. identified 41 unique metabolites associated with coffee consumption, of which O-methyl catechol sulfate and 3-methyl catechol sulfate were significantly and positively associated with CKD risk (41). Another Mendelian randomization study found coffee intake, particularly instant coffee, have an important role in shortening telomere length (42). Studies have shown that patients with advanced nephropathy in type 1 diabetes have faster telomere shortening than patients with non-advanced nephropathy. Similar situation is also found in in type 2 diabetes (43). So, coffee seems to be a risk factor for diabetic nephropathy, which was consistent with the results of our Mendelian randomization study.

Due to the drug resistance, adverse effects, and even toxicity of antidiabetic drugs, dietetic therapy is a new direction in treating diabetes (44). In recent years, there has been a proliferation of studies on the relationship between coffee consumption and the progression of diabetes and chronic kidney disease. Several short-term randomized controlled trials and epidemiological studies have explored the association between coffee consumption and the risk of T2DM. These studies have shown that heavy coffee consumption improves glucose tolerance and reduces the risk of T2DM (18, 45). Recently, a meta-analysis of cohort studies on Coffee consumption and mortality in patients with type 2 diabetes illustrated inverse associations between coffee consumption and the risk of mortality from all-cause, CVD, and CHD (46). Less than five studies were available for the analyses of mortality in patients with type 2 diabetes, thus leading to some confounding factors, selection bias, and information bias in the traditional epidemiologic study and making the relationship between coffee intake and diabetes remains elusive and needs further exploration. The causal link between coffee and diabetic nephropathy is still debatable because of the variety of diabetic nephropathy pathogenesis that is impacted by consumption of coffee. The primary cause of this disagreement may be the difficulty in obtaining deterministic causation in perspective and cross-sectional research due to confounding variables, including environment and selection bias.

Our present study reported a positive association between coffee intake and the risk of diabetic nephropathy, which is a significant cause of chronic kidney diseases in the elderly. There are several plausible mechanisms. First of all, caffeine is a widely recognised stimulant of the central nervous system and a significant xanthine alkaloid present in many common drinks, especially coffee (Coffea arabica and Coffea) (46). James et al. reported in a controlled clinical trial that regular caffeine consumption raises chronic glucose levels. Additionally, caffeine abstinence may enhance normal glucose control in patients with type 2 diabetes who consume daily coffee (47). Additionally, according to a cohort study, excessive coffee consumption was associated with albuminuria 2.8 years earlier than minimal coffee consumption (40). More than 95% of caffeine is metabolised by cytochrome P450 1A2 (CYP1A2), and a common CYP1A2 gene polymorphism has been linked to caffeine metabolism (48). Those with the AC and CC genotypes of CYP1A2 at rs762551, which are associated with a sluggish metabolism of caffeine, will be more at risk for albuminuria and hyperfiltration with heavy coffee intake (40). So it is of vital importance to reduce the risk of diabetic nephropathy with DNA-based interventions, such as precision nutrition recommendations. Moreover, caffeine is an adenosine receptor antagonist. When ingested, it binds to adenosine receptors (49–51) and interfered with adenosine’s anti-inflammatory and glomerular hemodynamic effects, resulting in albuminuria, glomerular remodelling and sclerosis (26, 27). In addition, most coffee drinks and instant coffee are loaded with refined sugars or coffee mate, which can lead to elevated blood sugar and hyperkalemia over time. Potassium is considered by influencing the body renal interstitial fibrosis and blood pressure level, oxidative stress and poor nutrition, such as mechanism, and hyperkalemia and hypokalemia short-term cause arrhythmia, indirect influence on the prognosis of patients with diabetic (52, 53).

Mendelian randomization (MR) is a statistical technique that uses genetic variants as proxies for modifiable risk factors to test causal relationships between a risk factor and an outcome, which may surmount some of the limitations inherent in traditional epidemiologic studies (54). As far as we’re aware, the present study is the first large-scale Mendelian randomization study of coffee intake and diabetic nephropathy. To address the bias issue, this study chose the GWAS data set with the most notable coffee intake and diabetic nephropathy participants. Then it examined 38 SNPs.3SNPs were excluded with MR-PRESSO outlier test *(P*<1) and 2 SNPs were excluded with PhenoScanner V2 to reduce bias and guarantee the reliability and validity of MR analysis. The IVW method showed that coffee intake increase was causally associated with a substantial increase in the risk of diabetic nephropathy especially type 2 diabetes. Multiple MR estimates and sensitivity analyses, including MR Egger, Cochran’s Q test, MR-PRESSO, and leave-one-out analysis, demonstrated that the results were reliable.

There were several strengths to our study. Firstly, the major merit is the MR design, which can help reduce the risk of confounding and reverse causal association, a significant limitation of observational studies. Secondly, we examined these correlations in 5 independent populations, and the consistent results ensured the reliability and validity of the study. In addition, several shortcomings also exist in this study. First, our study had a relatively small sample size and event number compared to population-based observational research although we selected the GWAS dataset with the largest sample size and the latest date in our MR analysis. Secondly, the population of individuals we included was all European ancestries. There is a population bias in the study, and the results do not applied to the whole people. The causal relationship between coffee intake and diabetic nephropathy should be further investigated with the inclusion of more pedigree populations. Thirdly, demographic characteristics and detailed clinical information about the included population were unavailable in the GWAS database, and subgroup analysis could not be performed statistically due to the insufficiency of the data. Subgroup analysis of the coffee intake, for example, <1 cup per day, 1-5cups per day, and> 5 cups per day, must be further considered for a more accurate and reliable conclusion. In addition, the exposure and outcome data may have substantial sample overlap, which could lead the model to be overfitted and the estimate of causality to be too heavily towards observational relationships. And MR analysis implies a linear relationship between exposure and outcome, which may not be accurate in all cases, limiting the method’s ability to detect non-linear effects and the interpretation of the results.

## Conclusion

To summarize, our MR analysis revealed a positive correlation between coffee intake and the risk of diabetic nephropathy from a limited perspective, corresponding to previous investigations that have described a crucial biological role for coffee in the progression of kidney dysfunction. In-depth and finer-scale whole-genome sequencing is needed better to characterize the causality between coffee intake and diabetic nephropathy. In the meantime, we look forward to conducting prospective, multicenter, large-sample, randomized controlled trials with longer follow-up time to clarify this association as soon as possible and achieving individualized treatment and precision medicine in diet therapy of diabetic nephropathy.

## Data availability statement

The original contributions presented in the study are included in the article/**Supplementary Material**. Further inquiries can be directed to the corresponding authors.

## Ethics statement

A publicly available GWAS catalog was used to conduct a two-sample MR study. Due to the re-analysis of previously summarized data, no additional ethical approval was required.

## Author contributions

JF and KS drafted the protocol, performed MR analysis and wrote the final paper. LY, DZ and HZ contributed to figure and table drawing and interpretation of results. JJ and QH contributed to the analysis and interpretation of data, as well as the critical review of the manuscript’s intellectually significant content. Each author contributed to the article and authorized the version that was submitted. All authors contributed to the article and approved the submitted version.
