# Corrigendum: Coffee intake and risk of diabetic nephropathy: a Mendelian randomization study

**DOI:** 10.3389/fendo.2023.1285535

**Published:** 2023-09-14

**Authors:** Jiaxi Fang, Kai Song, Di Zhang, Yan Liang, Huan Zhao, Juan Jin, Qiang He

**Affiliations:** ^1^ Urology & Nephrology Center, Department of Nephrology, Zhejiang Provincial People’s Hospital (Affiliated People’s Hospital, Hangzhou Medical College), Hangzhou, Zhejiang, China; ^2^ Zhejiang Provincial People’s Hospital, Qingdao University, Hangzhou, Zhejiang, China; ^3^ Geriatric Medicine Center, Department of Endocrinology, Zhejiang Provincial People’s Hospital, Affiliated People’s Hospital, Hangzhou Medical College, Hangzhou, Zhejiang, China; ^4^ Department of Nephrology, The First Affiliated Hospital of Zhejiang Chinese Medical University, Zhejiang Provincial Hospital of Traditional Chinese Medicine, Hangzhou, Zhejiang, China

**Keywords:** coffee intake, diabetic nephropathy, Mendelian randomization, causality, risk

**Incorrect Affiliation**

In the published article, there was an error in affiliations 1,2. Instead of “1 Zhejiang Provincial People’s Hospital, Qingdao University, Hangzhou, Zhejiang, China, 2 Nephrology Center, Department of Nephrology, Zhejiang Provincial People’s Hospital, Affiliated People’s Hospital, Hangzhou Medical College, Hangzhou, Zhejiang, China”, it should be “1 Urology & Nephrology Center, Department of Nephrology, Zhejiang Provincial People’s Hospital (Affiliated People’s Hospital, Hangzhou Medical College), Hangzhou, Zhejiang, China, 2 Zhejiang Provincial People’s Hospital, Qingdao University, Hangzhou, Zhejiang, China”.

The authors apologize for this error and state that this does not change the scientific conclusions of the article in any way. The original article has been updated.

