# Supplementary material for: Coffee intake and risk of diabetic nephropathy: a Mendelian randomization study
Source: Front Endocrinol (Lausanne). 2023 Jul 4;14:1169933. doi: 10.3389/fendo.2023.1169933 (PMC10352828; doi:10.3389/fendo.2023.1169933)
Supplement: Supplementary file 1 [file Image_1.pdf]

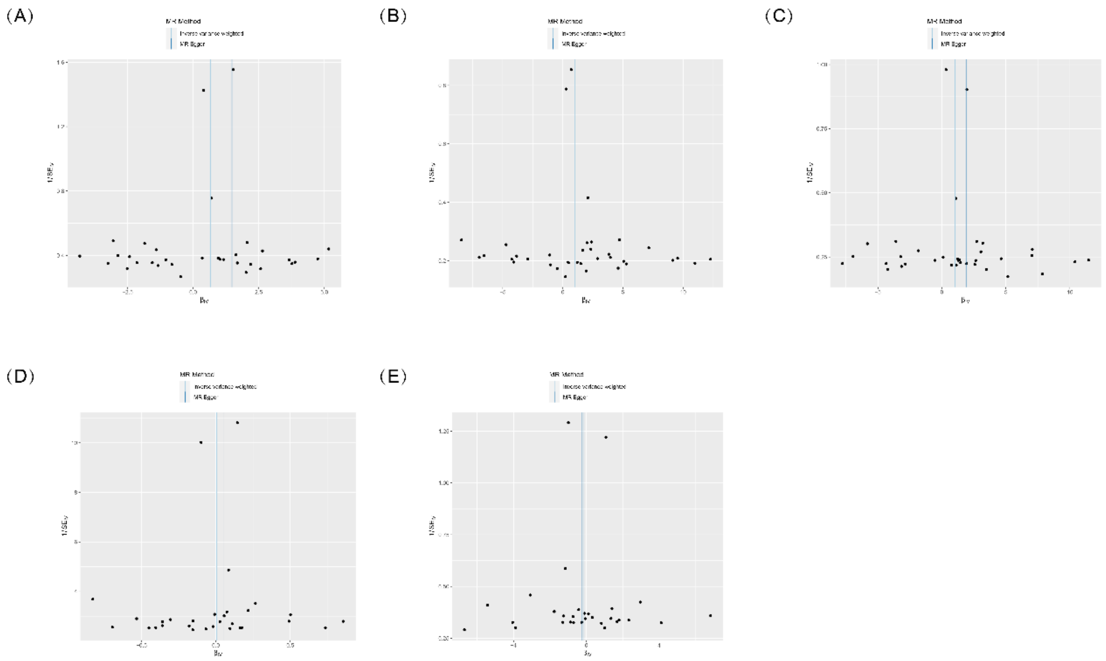

Supplementary Figure 1. Funnel plot showing heterogeneity on coffee intake and Diabetic nephropathy(A), type 1 diabetes with renal complications(B), type2 diabetes with renal complications(C), glomerular filtration rate in diabetics (D), urinary albumin-to-creatinine ratio(E)

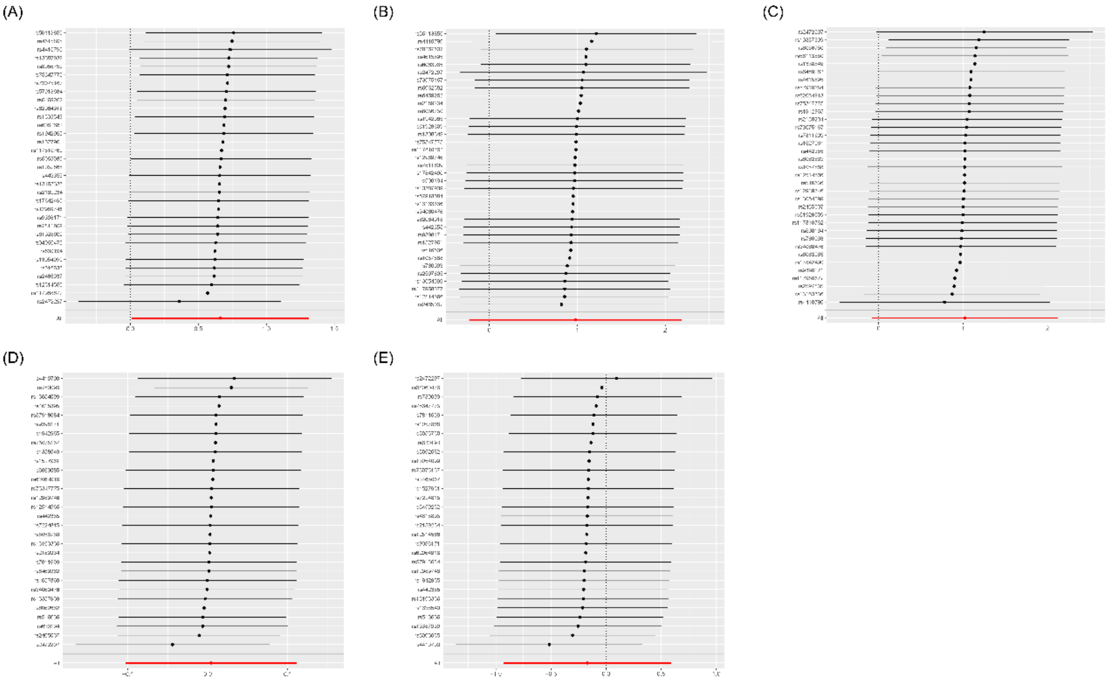

Supplementary Figure 2 Results of leave-one-out sensitivity analysis between each coffee intake SNP and Diabetic nephropathy(A), type 1 diabetes with renal complications(B), type2 diabetes with renal complications(C), glomerular filtration rate in diabetics (D), urinary albumin-to-creatinine ratio(E).
