# Supplementary material for: Coffee intake and risk of diabetic nephropathy: a Mendelian randomization study
Source: Front Endocrinol (Lausanne). 2023 Jul 4;14:1169933. doi: 10.3389/fendo.2023.1169933 (PMC10352828; doi:10.3389/fendo.2023.1169933)
Supplement: Supplementary file 2 [file Table_1.docx]

**Table S1** Characteristics of instrumental variables for Coffee intake

|  | **SNP** | **EA** | **OA** | **Samplesize** | **SE** | **β** | **id.exposure** | **EAF** | ***P* value** | **R^2^** | **F - statistic** |
| --- | --- | --- | --- | --- | --- | --- | --- | --- | --- | --- | --- |
| 1 | rs516636 | A | C | 428860 | 0.001984 | 0.0116767 | ukb-b-5237 | 0.208913 | 4.00E-09 | 4.50671E-05 | 19.32827262 |
| 2 | rs4615895 | A | G | 428860 | 0.00185 | 0.0122025 | ukb-b-5237 | 0.740926 | 4.20E-11 | 5.71645E-05 | 24.51684812 |
| 3 | rs13387939 | A | C | 428860 | 0.002139 | 0.0165558 | ukb-b-5237 | 0.828363 | 9.80E-15 | 7.79403E-05 | 33.42791675 |
| 4 | rs780093 | C | T | 428860 | 0.001657 | 0.0132935 | ukb-b-5237 | 0.615839 | 1.00E-15 | 8.3616E-05 | 35.86236848 |
| 5 | rs12989746 | T | G | 428860 | 0.001864 | 0.01035 | ukb-b-5237 | 0.249928 | 2.80E-08 | 4.01632E-05 | 17.22501155 |
| 6 | rs1527961 | C | T | 428860 | 0.002366 | -0.0133431 | ukb-b-5237 | 0.1349 | 1.70E-08 | 4.15549E-05 | 17.82187127 |
| 7 | rs2597805 | T | C | 428860 | 0.001756 | 0.00985502 | ukb-b-5237 | 0.682463 | 2.00E-08 | 4.20938E-05 | 18.05303655 |
| 8 | rs2189234 | G | T | 428860 | 0.001661 | 0.00998689 | ukb-b-5237 | 0.617795 | 1.80E-09 | 4.71011E-05 | 20.20064581 |
| 9 | rs13163336 | A | C | 428860 | 0.00221 | 0.0149472 | ukb-b-5237 | 0.15761 | 1.30E-11 | 5.93262E-05 | 25.44403242 |
| 10 | rs12514566 | A | G | 428860 | 0.001706 | -0.0113972 | ukb-b-5237 | 0.337107 | 2.40E-11 | 5.80547E-05 | 24.89867679 |
| 11 | rs2465037 | A | C | 428860 | 0.001707 | -0.0106317 | ukb-b-5237 | 0.343017 | 4.80E-10 | 5.09454E-05 | 21.84946667 |
| 12 | rs1338549 | G | T | 428860 | 0.001622 | 0.00945121 | ukb-b-5237 | 0.533932 | 5.60E-09 | 4.4457E-05 | 19.06658356 |
| 13 | rs9398171 | T | C | 428860 | 0.00178 | 0.0108577 | ukb-b-5237 | 0.71064 | 1.10E-09 | 4.84835E-05 | 20.79353792 |
| 14 | rs73075167 | T | A | 428860 | 0.002444 | -0.0160639 | ukb-b-5237 | 0.12918 | 5.00E-11 | 5.80571E-05 | 24.89971551 |
| 15 | rs7811609 | T | C | 428860 | 0.001665 | 0.00913864 | ukb-b-5237 | 0.374746 | 4.00E-08 | 3.91369E-05 | 16.78483717 |
| 16 | rs1057868 | T | C | 428860 | 0.001785 | 0.0199509 | ukb-b-5237 | 0.284986 | 5.40E-29 | 0.000162216 | 69.57881395 |
| 17 | rs4410790 | C | T | 428860 | 0.001673 | 0.039072 | ukb-b-5237 | 0.632141 | 1.20E-120 | 0.000709997 | 304.7043108 |
| 18 | rs34060476 | G | A | 428860 | 0.00237 | 0.0184292 | ukb-b-5237 | 0.133855 | 7.50E-15 | 7.87532E-05 | 33.77659624 |
| 19 | rs6469262 | C | T | 428860 | 0.001629 | -0.00915347 | ukb-b-5237 | 0.564966 | 1.90E-08 | 4.11858E-05 | 17.66356808 |
| 20 | rs78267637 | G | C | 428860 | 0.004317 | -0.0254259 | ukb-b-5237 | 0.038115 | 3.90E-09 | 4.74026E-05 | 20.32992817 |
| 21 | rs442355 | C | G | 428860 | 0.001854 | -0.0111372 | ukb-b-5237 | 0.254435 | 1.90E-09 | 4.70592E-05 | 20.18265757 |
| 22 | rs10119174 | C | G | 428860 | 0.001642 | -0.00939786 | ukb-b-5237 | 0.571035 | 1.00E-08 | 4.32686E-05 | 18.55687456 |
| 23 | rs117810762 | A | G | 428860 | 0.006179 | 0.0359086 | ukb-b-5237 | 0.017881 | 6.20E-09 | 4.5288E-05 | 19.42298805 |
| 24 | rs61928609 | C | A | 428860 | 0.002175 | -0.0147305 | ukb-b-5237 | 0.835328 | 1.30E-11 | 5.96955E-05 | 25.60243156 |
| 25 | rs2472297 | T | C | 428860 | 0.001827 | 0.0464708 | ukb-b-5237 | 0.262883 | 1.10E-142 | 0.00083693 | 359.2248445 |
| 26 | rs117968677 | A | G | 428860 | 0.005516 | -0.0310299 | ukb-b-5237 | 0.024207 | 1.90E-08 | 4.54872E-05 | 19.50844652 |
| 27 | rs8056750 | T | C | 428860 | 0.001737 | 0.0105333 | ukb-b-5237 | 0.359129 | 1.30E-09 | 5.10717E-05 | 21.90360986 |
| 28 | rs1421085 | C | T | 428860 | 0.001644 | 0.0185426 | ukb-b-5237 | 0.40357 | 1.70E-29 | 0.00016552 | 70.99618564 |
| 29 | rs62064918 | T | C | 428860 | 0.001879 | -0.0103075 | ukb-b-5237 | 0.244545 | 4.10E-08 | 3.92558E-05 | 16.835834 |
| 30 | rs57918684 | A | G | 428860 | 0.002238 | 0.0128864 | ukb-b-5237 | 0.154747 | 8.60E-09 | 4.34412E-05 | 18.63093082 |
| 31 | rs7224815 | T | A | 428860 | 0.001642 | -0.0108602 | ukb-b-5237 | 0.407832 | 3.70E-11 | 5.69681E-05 | 24.43262525 |
| 32 | rs630194 | C | T | 428860 | 0.001699 | -0.0113533 | ukb-b-5237 | 0.343374 | 2.30E-11 | 5.81246E-05 | 24.92863312 |
| 33 | rs1942965 | C | T | 428860 | 0.001619 | -0.00890339 | ukb-b-5237 | 0.504585 | 3.80E-08 | 3.96318E-05 | 16.99710692 |
| 34 | rs476828 | C | T | 14267 | 0.001895 | 0.0173461 | ukb-b-5237 | 0.237409 | 5.60E-20 | 0.000108949 | 46.72866521 |
| 35 | rs56113850 | C | T | 14267 | 0.001634 | 0.0126667 | ukb-b-5237 | 0.578109 | 8.90E-15 | 7.82649E-05 | 33.56714958 |
| 36 | rs75347775 | A | G | 14267 | 0.001879 | 0.0104504 | ukb-b-5237 | 0.244531 | 2.70E-08 | 4.03503E-05 | 17.30523238 |
| 37 | rs6063085 | C | A | 14267 | 0.001669 | 0.0104106 | ukb-b-5237 | 0.373473 | 4.50E-10 | 5.07201E-05 | 21.75284482 |
| 38 | rs6062682 | T | C | 14267 | 0.001639 | 0.0103704 | ukb-b-5237 | 0.464546 | 2.50E-10 | 5.35022E-05 | 22.94608807 |
| 39 | rs13054099 | C | T | 14267 | 0.001836 | -0.0107777 | ukb-b-5237 | 0.261004 | 4.30E-09 | 4.48096E-05 | 19.21783255 |
| 40 | rs17842490 | G | A | 14267 | 0.006808 | -0.0451683 | ukb-b-5237 | 0.014248 | 3.30E-11 | 5.73085E-05 | 24.57861829 |

SNP:single nucleotide polymorphisms; EA: effect allele; OA: other allele; EAF: effect allele frequency; SE, standard error

**Table S2.1** SNPs from GWAS on Coffee intake and Diabetic nephropathy

|  | | | **Exposure (Coffee intake)** | | |  | **Outcome (Diabetic nephropathy)** | | | | |
| --- | --- | --- | --- | --- | --- | --- | --- | --- | --- | --- | --- |
| **SNP** | **EA** | **OA** | **β** | **SE** | ***P* value** |  | **Case** | **Control** | **β** | **SE** | ***P* value** |
| rs1057868 | T | C | 0.019951 | 0.001785 | 5.40E-29 |  | 2,026 | 203,018 | 0.0341 | 0.0334 | 0.3077 |
| rs117810762 | A | G | 0.035909 | 0.006179 | 6.20E-09 |  | 2,026 | 203,018 | -0.2181 | 0.1748 | 0.2122 |
| rs117968677 | A | G | -0.03103 | 0.005516 | 1.90E-08 |  | 2,026 | 203,018 | 0.0919 | 0.088 | 0.2966 |
| rs12514566 | A | G | -0.0114 | 0.001706 | 2.40E-11 |  | 2,026 | 203,018 | -0.0425 | 0.0381 | 0.2648 |
| rs12989746 | T | G | 0.01035 | 0.001864 | 2.80E-08 |  | 2,026 | 203,018 | -0.0277 | 0.0373 | 0.457 |
| rs13054099 | C | T | -0.01078 | 0.001836 | 4.30E-09 |  | 2,026 | 203,018 | -0.0046 | 0.0393 | 0.9066 |
| rs13163336 | A | C | 0.014947 | 0.00221 | 1.30E-11 |  | 2,026 | 203,018 | -0.0112 | 0.0505 | 0.8246 |
| rs1338549 | G | T | -0.00945 | 0.001622 | 5.60E-09 |  | 2,026 | 203,018 | 0.0508 | 0.0338 | 0.1328 |
| rs13387939 | A | C | 0.016556 | 0.002139 | 9.80E-15 |  | 2,026 | 203,018 | 0.0343 | 0.0444 | 0.4393 |
| rs17842490 | G | A | -0.04517 | 0.006808 | 3.30E-11 |  | 2,026 | 203,018 | -0.1665 | 0.1966 | 0.3972 |
| rs1942965 | C | T | -0.0089 | 0.001619 | 3.80E-08 |  | 2,026 | 203,018 | 0.0323 | 0.0333 | 0.332 |
| rs2189234 | G | T | 0.009987 | 0.001661 | 1.80E-09 |  | 2,026 | 203,018 | -0.0206 | 0.0338 | 0.5433 |
| rs2465037 | A | C | -0.01063 | 0.001707 | 4.80E-10 |  | 2,026 | 203,018 | 0.0212 | 0.0361 | 0.557799 |
| rs2472297 | T | C | 0.046471 | 0.001827 | 1.10E-142 |  | 2,026 | 203,018 | 0.0252 | 0.0381 | 0.5092 |
| rs2597805 | T | C | 0.009855 | 0.001756 | 2.00E-08 |  | 2,026 | 203,018 | -6.00E-04 | 0.0342 | 0.9866 |
| rs34060476 | G | A | 0.018429 | 0.00237 | 7.50E-15 |  | 2,026 | 203,018 | 0.026 | 0.0482 | 0.588901 |
| rs4410790 | C | T | 0.039072 | 0.001673 | 1.20E-120 |  | 2,026 | 203,018 | 0.0652 | 0.0347 | 0.0601797 |
| rs442355 | C | G | -0.01114 | 0.001854 | 1.90E-09 |  | 2,026 | 203,018 | -0.0456 | 0.0369 | 0.2167 |
| rs4615895 | A | G | 0.012203 | 0.00185 | 4.20E-11 |  | 2,026 | 203,018 | 0.0098 | 0.0391 | 0.802 |
| rs516636 | A | C | 0.011677 | 0.001984 | 4.00E-09 |  | 2,026 | 203,018 | -0.0041 | 0.0412 | 0.9204 |
| rs56113850 | C | T | 0.012667 | 0.001634 | 8.90E-15 |  | 2,026 | 203,018 | 0.0015 | 0.0327 | 0.9628 |
| rs57918684 | A | G | 0.012886 | 0.002238 | 8.60E-09 |  | 2,026 | 203,018 | 0.0077 | 0.0376 | 0.8367 |
| rs6062682 | T | C | 0.01037 | 0.001639 | 2.50E-10 |  | 2,026 | 203,018 | 0.0291 | 0.0353 | 0.4107 |
| rs6063085 | C | A | 0.010411 | 0.001669 | 4.50E-10 |  | 2,026 | 203,018 | -0.0411 | 0.0344 | 0.2315 |
| rs61928609 | C | A | -0.01473 | 0.002175 | 1.30E-11 |  | 2,026 | 203,018 | -0.045 | 0.0589 | 0.4441 |
| rs62064918 | T | C | -0.01031 | 0.001879 | 4.10E-08 |  | 2,026 | 203,018 | -0.0041 | 0.0411 | 0.9196 |
| rs630194 | C | T | -0.01135 | 0.001699 | 2.30E-11 |  | 2,026 | 203,018 | -0.0433 | 0.0336 | 0.1979 |
| rs73075167 | T | A | -0.01606 | 0.002444 | 5.00E-11 |  | 2,026 | 203,018 | 0.0187 | 0.0523 | 0.7209 |
| rs75347775 | A | G | 0.01045 | 0.001879 | 2.70E-08 |  | 2,026 | 203,018 | 0.0451 | 0.0378 | 0.2329 |
| rs780093 | C | T | 0.013294 | 0.001657 | 1.00E-15 |  | 2,026 | 203,018 | 0.0319 | 0.0343 | 0.3531 |
| rs7811609 | T | C | 0.009139 | 0.001665 | 4.00E-08 |  | 2,026 | 203,018 | -0.0109 | 0.0336 | 0.7465 |
| rs78267637 | G | C | -0.02543 | 0.004317 | 3.90E-09 |  | 2,026 | 203,018 | 0.0031 | 0.0701 | 0.9651 |
| rs8056750 | T | C | 0.010533 | 0.001737 | 1.30E-09 |  | 2,026 | 203,018 | -0.0157 | 0.0335 | 0.6391 |
| rs9398171 | T | C | 0.010858 | 0.00178 | 1.10E-09 |  | 2,026 | 203,018 | 0.023 | 0.0342 | 0.500299 |
| rs6469262 | C | T | -0.00915 | 0.001629 | 1.90E-08 |  | 2,026 | 203,018 | 0.0739 | 0.0328 | 0.02442 |
| rs1527961 | C | T | -0.01334 | 0.002366 | 1.70E-08 |  | 2,026 | 203,018 | -0.107 | 0.0496 | 0.03086 |

SNP:single nucleotide polymorphisms; EA: effect allele; OA: other allele; SE, standard error

**Table S2.2** SNPs from GWAS on Coffee intake and Type 1 diabetes with renal complications

|  | | | **Exposure (Coffee intake)** | | |  | **Outcome (Type 1 diabetes with renal complications)** | | | | |
| --- | --- | --- | --- | --- | --- | --- | --- | --- | --- | --- | --- |
| **SNP** | **EA** | **OA** | **β** | **SE** | ***P* value** |  | **Case** | **Control** | **β** | **SE** | ***P* value** |
| rs10119174 | C | G | -0.0094 | 0.001642 | 1.00E-08 |  | 963 | 183,185 | -0.061 | 0.0501 | 0.2231 |
| rs1057868 | T | C | 0.019951 | 0.001785 | 5.40E-29 |  | 963 | 183,185 | 0.0415 | 0.048 | 0.388 |
| rs117810762 | A | G | 0.035909 | 0.006179 | 6.20E-09 |  | 963 | 183,185 | 0.0077 | 0.2472 | 0.975 |
| rs117968677 | A | G | -0.03103 | 0.005516 | 1.90E-08 |  | 963 | 183,185 | -0.2218 | 0.1272 | 0.0811708 |
| rs12514566 | A | G | -0.0114 | 0.001706 | 2.40E-11 |  | 963 | 183,185 | -0.1088 | 0.0547 | 0.0468997 |
| rs12989746 | T | G | 0.01035 | 0.001864 | 2.80E-08 |  | 963 | 183,185 | 0.0124 | 0.0532 | 0.8154 |
| rs13054099 | C | T | -0.01078 | 0.001836 | 4.30E-09 |  | 963 | 183,185 | -0.1184 | 0.0563 | 0.0355697 |
| rs13163336 | A | C | 0.014947 | 0.00221 | 1.30E-11 |  | 963 | 183,185 | 0.0431 | 0.0721 | 0.5498 |
| rs1338549 | G | T | -0.00945 | 0.001622 | 5.60E-09 |  | 963 | 183,185 | -0.0042 | 0.0485 | 0.9305 |
| rs13387939 | A | C | 0.016556 | 0.002139 | 9.80E-15 |  | 963 | 183,185 | 0.0333 | 0.0633 | 0.5992 |
| rs1527961 | C | T | -0.01334 | 0.002366 | 1.70E-08 |  | 963 | 183,185 | -0.0705 | 0.0706 | 0.3183 |
| rs17842490 | G | A | -0.04517 | 0.006808 | 3.30E-11 |  | 963 | 183,185 | -0.0877 | 0.2739 | 0.7489 |
| rs1942965 | C | T | -0.0089 | 0.001619 | 3.80E-08 |  | 963 | 183,185 | 0.0092 | 0.0479 | 0.8474 |
| rs2189234 | G | T | 0.009987 | 0.001661 | 1.80E-09 |  | 963 | 183,185 | -0.0291 | 0.0485 | 0.548001 |
| rs2465037 | A | C | -0.01063 | 0.001707 | 4.80E-10 |  | 963 | 183,185 | -0.1305 | 0.0519 | 0.01187 |
| rs2472297 | T | C | 0.046471 | 0.001827 | 1.10E-142 |  | 963 | 183,185 | 0.0324 | 0.0544 | 0.5512 |
| rs2597805 | T | C | 0.009855 | 0.001756 | 2.00E-08 |  | 963 | 183,185 | 0.0901 | 0.0489 | 0.0650399 |
| rs34060476 | G | A | 0.018429 | 0.00237 | 7.50E-15 |  | 963 | 183,185 | 0.0439 | 0.0698 | 0.5295 |
| rs4410790 | C | T | 0.039072 | 0.001673 | 1.20E-120 |  | 963 | 183,185 | 0.0107 | 0.0496 | 0.8292 |
| rs442355 | C | G | -0.01114 | 0.001854 | 1.90E-09 |  | 963 | 183,185 | -0.044 | 0.0527 | 0.4042 |
| rs4615895 | A | G | 0.012203 | 0.00185 | 4.20E-11 |  | 963 | 183,185 | -0.08 | 0.0562 | 0.1542 |
| rs516636 | A | C | 0.011677 | 0.001984 | 4.00E-09 |  | 963 | 183,185 | 0.0591 | 0.059 | 0.3172 |
| rs56113850 | C | T | 0.012667 | 0.001634 | 8.90E-15 |  | 963 | 183,185 | -0.1068 | 0.0467 | 0.0222398 |
| rs57918684 | A | G | 0.012886 | 0.002238 | 8.60E-09 |  | 963 | 183,185 | 0.0299 | 0.0538 | 0.5781 |
| rs6062682 | T | C | 0.01037 | 0.001639 | 2.50E-10 |  | 963 | 183,185 | -0.044 | 0.0506 | 0.3842 |
| rs6063085 | C | A | 0.010411 | 0.001669 | 4.50E-10 |  | 963 | 183,185 | -0.0724 | 0.0493 | 0.142 |
| rs61928609 | C | A | -0.01473 | 0.002175 | 1.30E-11 |  | 963 | 183,185 | 0.0069 | 0.0851 | 0.9354 |
| rs62064918 | T | C | -0.01031 | 0.001879 | 4.10E-08 |  | 963 | 183,185 | -0.0474 | 0.0591 | 0.4225 |
| rs630194 | C | T | -0.01135 | 0.001699 | 2.30E-11 |  | 963 | 183,185 | -0.0187 | 0.0481 | 0.697199 |
| rs6469262 | C | T | -0.00915 | 0.001629 | 1.90E-08 |  | 963 | 183,185 | 0.0374 | 0.0469 | 0.4249 |
| rs7224815 | T | A | -0.01086 | 0.001642 | 3.70E-11 |  | 963 | 183,185 | -0.031 | 0.0469 | 0.5081 |
| rs73075167 | T | A | -0.01606 | 0.002444 | 5.00E-11 |  | 963 | 183,185 | 0.0617 | 0.0744 | 0.4072 |
| rs75347775 | A | G | 0.01045 | 0.001879 | 2.70E-08 |  | 963 | 183,185 | 0.0049 | 0.0541 | 0.9272 |
| rs780093 | C | T | 0.013294 | 0.001657 | 1.00E-15 |  | 963 | 183,185 | 0.0626 | 0.049 | 0.2017 |
| rs7811609 | T | C | 0.009139 | 0.001665 | 4.00E-08 |  | 963 | 183,185 | 0.0136 | 0.0481 | 0.7778 |
| rs78267637 | G | C | -0.02543 | 0.004317 | 3.90E-09 |  | 963 | 183,185 | 0.1199 | 0.0997 | 0.2292 |
| rs8056750 | T | C | 0.010533 | 0.001737 | 1.30E-09 |  | 963 | 183,185 | -0.0115 | 0.048 | 0.8109 |
| rs9398171 | T | C | 0.010858 | 0.00178 | 1.10E-09 |  | 963 | 183,185 | 0.0415 | 0.0489 | 0.3961 |

SNP:single nucleotide polymorphisms; EA: effect allele; OA: other allele; SE, standard error

**Table S2.3** SNPs from GWAS on Coffee intake and Type2 diabetes with renal complications

|  | | | **Exposure (Coffee intake)** | | |  | **Outcome (Type2 diabetes with renal complications)** | | | | |
| --- | --- | --- | --- | --- | --- | --- | --- | --- | --- | --- | --- |
| **SNP** | **EA** | **OA** | **β** | **SE** | ***P* value** |  | **Case** | **Control** | **β** | **SE** | ***P* value** |
| rs10119174 | C | G | -0.0094 | 0.001642 | 1.00E-08 |  | 1,296 | 183,185 | -0.0568 | 0.0433 | 0.1893 |
| rs1057868 | T | C | 0.019951 | 0.001785 | 5.40E-29 |  | 1,296 | 183,185 | 0.0221 | 0.0417 | 0.597 |
| rs117810762 | A | G | 0.035909 | 0.006179 | 6.20E-09 |  | 1,296 | 183,185 | 0.1851 | 0.2077 | 0.3727 |
| rs117968677 | A | G | -0.03103 | 0.005516 | 1.90E-08 |  | 1,296 | 183,185 | -0.2192 | 0.1111 | 0.04844 |
| rs12514566 | A | G | -0.0114 | 0.001706 | 2.40E-11 |  | 1,296 | 183,185 | -0.0157 | 0.0477 | 0.7426 |
| rs12989746 | T | G | 0.01035 | 0.001864 | 2.80E-08 |  | 1,296 | 183,185 | 0.02 | 0.0462 | 0.6655 |
| rs13054099 | C | T | -0.01078 | 0.001836 | 4.30E-09 |  | 1,296 | 183,185 | -0.0278 | 0.0488 | 0.5683 |
| rs13163336 | A | C | 0.014947 | 0.00221 | 1.30E-11 |  | 1,296 | 183,185 | 0.1714 | 0.0629 | 0.006389 |
| rs1338549 | G | T | -0.00945 | 0.001622 | 5.60E-09 |  | 1,296 | 183,185 | 0.0734 | 0.0421 | 0.08124 |
| rs13387939 | A | C | 0.016556 | 0.002139 | 9.80E-15 |  | 1,296 | 183,185 | -0.0964 | 0.0549 | 0.07927 |
| rs1527961 | C | T | -0.01334 | 0.002366 | 1.70E-08 |  | 1,296 | 183,185 | -0.0151 | 0.0612 | 0.8047 |
| rs17842490 | G | A | -0.04517 | 0.006808 | 3.30E-11 |  | 1,296 | 183,185 | -0.3548 | 0.2464 | 0.1499 |
| rs1942965 | C | T | -0.0089 | 0.001619 | 3.80E-08 |  | 1,296 | 183,185 | 0.0281 | 0.0418 | 0.502 |
| rs2189234 | G | T | 0.009987 | 0.001661 | 1.80E-09 |  | 1,296 | 183,185 | -0.0053 | 0.0422 | 0.9005 |
| rs2465037 | A | C | -0.01063 | 0.001707 | 4.80E-10 |  | 1,296 | 183,185 | -0.0281 | 0.045 | 0.5327 |
| rs2472297 | T | C | 0.046471 | 0.001827 | ####### |  | 1,296 | 183,185 | 0.0153 | 0.0474 | 0.7473 |
| rs2597805 | T | C | 0.009855 | 0.001756 | 2.00E-08 |  | 1,296 | 183,185 | 0.1025 | 0.0427 | 0.01633 |
| rs34060476 | G | A | 0.018429 | 0.00237 | 7.50E-15 |  | 1,296 | 183,185 | 0.0589 | 0.0607 | 0.332 |
| rs4410790 | C | T | 0.039072 | 0.001673 | ####### |  | 1,296 | 183,185 | 0.0763 | 0.0433 | 0.078069 |
| rs442355 | C | G | -0.01114 | 0.001854 | 1.90E-09 |  | 1,296 | 183,185 | -0.0137 | 0.0459 | 0.764499 |
| rs4615895 | A | G | 0.012203 | 0.00185 | 4.20E-11 |  | 1,296 | 183,185 | -0.0392 | 0.0486 | 0.4204 |
| rs516636 | A | C | 0.011677 | 0.001984 | 4.00E-09 |  | 1,296 | 183,185 | 0.017 | 0.0513 | 0.740399 |
| rs56113850 | C | T | 0.012667 | 0.001634 | 8.90E-15 |  | 1,296 | 183,185 | -0.0455 | 0.0408 | 0.2642 |
| rs57918684 | A | G | 0.012886 | 0.002238 | 8.60E-09 |  | 1,296 | 183,185 | -0.0236 | 0.047 | 0.6147 |
| rs6062682 | T | C | 0.01037 | 0.001639 | 2.50E-10 |  | 1,296 | 183,185 | 0.0134 | 0.0439 | 0.7602 |
| rs6063085 | C | A | 0.010411 | 0.001669 | 4.50E-10 |  | 1,296 | 183,185 | 0.0482 | 0.0428 | 0.2603 |
| rs61928609 | C | A | -0.01473 | 0.002175 | 1.30E-11 |  | 1,296 | 183,185 | -0.0512 | 0.0732 | 0.4844 |
| rs62064918 | T | C | -0.01031 | 0.001879 | 4.10E-08 |  | 1,296 | 183,185 | 0.0436 | 0.0511 | 0.3937 |
| rs630194 | C | T | -0.01135 | 0.001699 | 2.30E-11 |  | 1,296 | 183,185 | -0.0346 | 0.042 | 0.4094 |
| rs6469262 | C | T | -0.00915 | 0.001629 | 1.90E-08 |  | 1,296 | 183,185 | 0.0398 | 0.0408 | 0.3294 |
| rs7224815 | T | A | -0.01086 | 0.001642 | 3.70E-11 |  | 1,296 | 183,185 | -0.042 | 0.0409 | 0.3043 |
| rs73075167 | T | A | -0.01606 | 0.002444 | 5.00E-11 |  | 1,296 | 183,185 | -0.0016 | 0.0646 | 0.9805 |
| rs75347775 | A | G | 0.01045 | 0.001879 | 2.70E-08 |  | 1,296 | 183,185 | -0.0299 | 0.047 | 0.524 |
| rs780093 | C | T | 0.013294 | 0.001657 | 1.00E-15 |  | 1,296 | 183,185 | 0.0362 | 0.0428 | 0.3977 |
| rs7811609 | T | C | 0.009139 | 0.001665 | 4.00E-08 |  | 1,296 | 183,185 | 0.007 | 0.0419 | 0.868 |
| rs8056750 | T | C | 0.010533 | 0.001737 | 1.30E-09 |  | 1,296 | 183,185 | -0.0731 | 0.0418 | 0.08064 |
| rs9398171 | T | C | 0.010858 | 0.00178 | 1.10E-09 |  | 1,296 | 183,185 | 0.0764 | 0.0425 | 0.072069 |

SNP:single nucleotide polymorphisms; EA: effect allele; OA: other allele; SE, standard error

**Table S2.4** SNPs from GWAS on Coffee intake and Glomerular filtration rate in diabetics

|  | | | **Exposure (Coffee intake)** | | |  | **Outcome (Glomerular filtration rate in diabetics)** | | | | |
| --- | --- | --- | --- | --- | --- | --- | --- | --- | --- | --- | --- |
| **SNP** | **EA** | **OA** | **β** | **SE** | ***P* value** |  | **Case** | **Control** | **β** | **SE** | ***P* value** |
| rs10119174 | C | G | -0.0094 | 0.00164159 | 1.00E-08 |  | 11,522 | 133,413 | -9.00E-04 | 0.0037 | 0.8 |
| rs1057868 | T | C | 0.019951 | 0.00178517 | 5.40E-29 |  | 11,522 | 133,413 | 0.0017 | 0.0041 | 0.68 |
| rs12514566 | A | G | -0.0114 | 0.00170562 | 2.40E-11 |  | 11,522 | 133,413 | 1.00E-04 | 0.0037 | 0.98 |
| rs12989746 | T | G | 0.01035 | 0.00186429 | 2.80E-08 |  | 11,522 | 133,413 | -2.00E-04 | 0.004 | 0.97 |
| rs13054099 | C | T | -0.01078 | 0.00183597 | 4.30E-09 |  | 11,522 | 133,413 | 0.0075 | 0.0042 | 0.0719996 |
| rs13163336 | A | C | 0.014947 | 0.00221005 | 1.30E-11 |  | 11,522 | 133,413 | 0.0011 | 0.0047 | 0.81 |
| rs1338549 | G | T | -0.00945 | 0.0016218 | 5.60E-09 |  | 11,522 | 133,413 | 0.0034 | 0.0036 | 0.34 |
| rs13387939 | A | C | 0.016556 | 0.0021386 | 9.80E-15 |  | 11,522 | 133,413 | 0.0044 | 0.0047 | 0.34 |
| rs1527961 | C | T | -0.01334 | 0.00236585 | 1.70E-08 |  | 11,522 | 133,413 | 0.0024 | 0.0051 | 0.64 |
| rs1942965 | C | T | -0.0089 | 0.00161917 | 3.80E-08 |  | 11,522 | 133,413 | 0.0036 | 0.0035 | 0.31 |
| rs2189234 | G | T | 0.009987 | 0.00166052 | 1.80E-09 |  | 11,522 | 133,413 | 0.0011 | 0.0037 | 0.760001 |
| rs2465037 | A | C | -0.01063 | 0.00170743 | 4.80E-10 |  | 11,522 | 133,413 | -0.0091 | 0.0038 | 0.016 |
| rs2472297 | T | C | 0.046471 | 0.00182733 | 1.10E-142 |  | 11,522 | 133,413 | 0.0067 | 0.0043 | 0.12 |
| rs34060476 | G | A | 0.018429 | 0.00237033 | 7.50E-15 |  | 11,522 | 133,413 | 0.004 | 0.0057 | 0.48 |
| rs4410790 | C | T | 0.039072 | 0.00167288 | 1.20E-120 |  | 11,522 | 133,413 | -0.0039 | 0.0039 | 0.32 |
| rs442355 | C | G | -0.01114 | 0.00185374 | 1.90E-09 |  | 11,522 | 133,413 | -3.00E-04 | 0.004 | 0.93 |
| rs4615895 | A | G | 0.012203 | 0.00184972 | 4.20E-11 |  | 11,522 | 133,413 | -0.0065 | 0.0042 | 0.12 |
| rs516636 | A | C | 0.011677 | 0.00198419 | 4.00E-09 |  | 11,522 | 133,413 | 0.0086 | 0.0046 | 0.061 |
| rs57918684 | A | G | 0.012886 | 0.00223845 | 8.60E-09 |  | 11,522 | 133,413 | -0.0058 | 0.0051 | 0.26 |
| rs6062682 | T | C | 0.01037 | 0.00163929 | 2.50E-10 |  | 11,522 | 133,413 | 0.0051 | 0.0037 | 0.16 |
| rs6063085 | C | A | 0.010411 | 0.00166919 | 4.50E-10 |  | 11,522 | 133,413 | -0.0016 | 0.0037 | 0.67 |
| rs62064918 | T | C | -0.01031 | 0.00187872 | 4.10E-08 |  | 11,522 | 133,413 | 0.0016 | 0.0042 | 0.7 |
| rs630194 | C | T | -0.01135 | 0.00169853 | 2.30E-11 |  | 11,522 | 133,413 | -0.0057 | 0.0037 | 0.13 |
| rs6469262 | C | T | -0.00915 | 0.00162895 | 1.90E-08 |  | 11,522 | 133,413 | -0.0016 | 0.0036 | 0.66 |
| rs7224815 | T | A | -0.01086 | 0.00164162 | 3.70E-11 |  | 11,522 | 133,413 | -6.00E-04 | 0.0036 | 0.87 |
| rs73075167 | T | A | -0.01606 | 0.00244429 | 5.00E-11 |  | 11,522 | 133,413 | 0.0049 | 0.0056 | 0.39 |
| rs75347775 | A | G | 0.01045 | 0.00187898 | 2.70E-08 |  | 11,522 | 133,413 | -7.00E-04 | 0.0042 | 0.87 |
| rs780093 | C | T | 0.013294 | 0.00165695 | 1.00E-15 |  | 11,522 | 133,413 | -0.011 | 0.0036 | 0.0022 |
| rs7811609 | T | C | 0.009139 | 0.00166468 | 4.00E-08 |  | 11,522 | 133,413 | 0.0015 | 0.0036 | 0.68 |
| rs8056750 | T | C | 0.010533 | 0.00173692 | 1.30E-09 |  | 11,522 | 133,413 | 0.001 | 0.0042 | 0.82 |
| rs9398171 | T | C | 0.010858 | 0.00177995 | 1.10E-09 |  | 11,522 | 133,413 | -0.0039 | 0.0039 | 0.32 |

SNP:single nucleotide polymorphisms; EA: effect allele; OA: other allele; SE, standard error

**Table S2.5** SNPs from GWAS on Coffee intake and albumin-to-creatinine ratio in diabetics

|  | | | **Exposure (Coffee intake)** | | |  | **Outcome (albumin-to-creatinine ratio in diabetics)** | | | | |
| --- | --- | --- | --- | --- | --- | --- | --- | --- | --- | --- | --- |
| **SNP** | **EA** | **OA** | **β** | **SE** | ***P* value** |  | **Case** | **Control** | **β** | **SE** | ***P* value** |
| rs10119174 | C | G | -0.00939786 | 0.001642 | 1.00E-08 |  | 5,825 | 46061 | -0.0048 | 0.029 | 0.87 |
| rs1057868 | T | C | 0.0199509 | 0.001785 | 5.40E-29 |  | 5,825 | 46061 | -0.023 | 0.034 | 0.5 |
| rs12514566 | A | G | -0.0113972 | 0.001706 | 2.40E-11 |  | 5,825 | 46061 | -0.0013 | 0.031 | 0.97 |
| rs12989746 | T | G | 0.01035 | 0.001864 | 2.80E-08 |  | 5,825 | 46061 | 0.014 | 0.03 | 0.649999 |
| rs13054099 | C | T | -0.0107777 | 0.001836 | 4.30E-09 |  | 5,825 | 46061 | 0.014 | 0.033 | 0.67 |
| rs13163336 | A | C | 0.0149472 | 0.00221 | 1.30E-11 |  | 5,825 | 46061 | 0.021 | 0.038 | 0.57 |
| rs1338549 | G | T | -0.00945121 | 0.001622 | 5.60E-09 |  | 5,825 | 46061 | -0.022 | 0.028 | 0.44 |
| rs13387939 | A | C | 0.0165558 | 0.002139 | 9.80E-15 |  | 5,825 | 46061 | 0.049 | 0.039 | 0.21 |
| rs1527961 | C | T | -0.0133431 | 0.002366 | 1.70E-08 |  | 5,825 | 46061 | 0.0094 | 0.041 | 0.82 |
| rs1942965 | C | T | -0.00890339 | 0.001619 | 3.80E-08 |  | 5,825 | 46061 | -0.015 | 0.027 | 0.58 |
| rs2189234 | G | T | 0.00998689 | 0.001661 | 1.80E-09 |  | 5,825 | 46061 | -0.0006 | 0.029 | 0.98 |
| rs2465037 | A | C | -0.0106317 | 0.001707 | 4.80E-10 |  | 5,825 | 46061 | 0.0078 | 0.03 | 0.79 |
| rs2472297 | T | C | 0.0464708 | 0.001827 | 1.10E-142 |  | 5,825 | 46061 | -0.046 | 0.036 | 0.2 |
| rs34060476 | G | A | 0.0184292 | 0.00237 | 7.50E-15 |  | 5,825 | 46061 | -0.1 | 0.045 | 0.022 |
| rs4410790 | C | T | 0.039072 | 0.001673 | 1.20E-120 |  | 5,825 | 46061 | 0.042 | 0.032 | 0.18 |
| rs442355 | C | G | -0.0111372 | 0.001854 | 1.90E-09 |  | 5,825 | 46061 | -0.02 | 0.033 | 0.54 |
| rs4615895 | A | G | 0.0122025 | 0.00185 | 4.20E-11 |  | 5,825 | 46061 | -0.0012 | 0.033 | 0.97 |
| rs516636 | A | C | 0.0116767 | 0.001984 | 4.00E-09 |  | 5,825 | 46061 | 0.048 | 0.036 | 0.18 |
| rs57918684 | A | G | 0.0128864 | 0.002238 | 8.60E-09 |  | 5,825 | 46061 | 0.013 | 0.043 | 0.77 |
| rs6062682 | T | C | 0.0103704 | 0.001639 | 2.50E-10 |  | 5,825 | 46061 | -0.013 | 0.029 | 0.66 |
| rs6063085 | C | A | 0.0104106 | 0.001669 | 4.50E-10 |  | 5,825 | 46061 | 0.071 | 0.029 | 0.013 |
| rs62064918 | T | C | -0.0103075 | 0.001879 | 4.10E-08 |  | 5,825 | 46061 | -0.0085 | 0.032 | 0.79 |
| rs630194 | C | T | -0.0113533 | 0.001699 | 2.30E-11 |  | 5,825 | 46061 | 0.02 | 0.03 | 0.51 |
| rs6469262 | C | T | -0.00915347 | 0.001629 | 1.90E-08 |  | 5,825 | 46061 | 0.0024 | 0.028 | 0.93 |
| rs7224815 | T | A | -0.0108602 | 0.001642 | 3.70E-11 |  | 5,825 | 46061 | 0.0046 | 0.028 | 0.87 |
| rs73075167 | T | A | -0.0160639 | 0.002444 | 5.00E-11 |  | 5,825 | 46061 | 0.014 | 0.049 | 0.77 |
| rs75347775 | A | G | 0.0104504 | 0.001879 | 2.70E-08 |  | 5,825 | 46061 | -0.07 | 0.036 | 0.054 |
| rs780093 | C | T | 0.0132935 | 0.001657 | 1.00E-15 |  | 5,825 | 46061 | -0.041 | 0.029 | 0.15 |
| rs7811609 | T | C | 0.00913864 | 0.001665 | 4.00E-08 |  | 5,825 | 46061 | -0.037 | 0.028 | 0.18 |
| rs8056750 | T | C | 0.0105333 | 0.001737 | 1.30E-09 |  | 5,825 | 46061 | -0.041 | 0.035 | 0.24 |
| rs9398171 | T | C | 0.0108577 | 0.00178 | 1.10E-09 |  | 5,825 | 46061 | 0.0035 | 0.031 | 0.91 |

SNP:single nucleotide polymorphisms; EA: effect allele; OA: other allele; SE, standard error

**Table S3** Heterogeneity of MR analysis for Coffee intake and Diabetic nephropathy

| **Exposure** | **Outcome** | **Method** | | **Q** | **Q_df** | **Q_P val** |
| --- | --- | --- | --- | --- | --- | --- |
| **Coffee intake** | Diabetic nephropathy | | MR Egger | 30.62383 | 31 | 0.4852777 |
| **Coffee intake** | Diabetic nephropathy | | Inverse variance weighted | 32.95208 | 32 | 0.07832620 |
| **Coffee intake** | Glomerular filtration rate in diabetics | | MR Egger | 39.16366 | 28 | 0.07832620 |
| **Coffee intake** | Glomerular filtration rate in diabetics | | Inverse variance weighted | 39.58254 | 29 | 0.09099741 |
| **Coffee intake** | Type2 diabetes with renal complications | | MR Egger | 40.89378 | 33 | 0.1625259 |
| **Coffee intake** | Type2 diabetes with renal complications | | Inverse variance weighted | 41.93710 | 34 | 0.1645560 |
| **Coffee intake** | Type 1 diabetes with renal complications | | MR Egger | 39.92045 | 34 | 0.2236522 |
| **Coffee intake** | Type 1 diabetes with renal complications | | Inverse variance weighted | 40.21583 | 35 | 0.2502408 |
| **Coffee intake** | albumin-to-creatinine ratio | | MR Egger | 29.84959 | 28 | 0.3704435 |
| **Coffee intake** | albumin-to-creatinine ratio | | Inverse variance weighted | 29.85761 | 29 | 0.4211374 |

**Table S4** The results of MR-Egger intercept analysis, MR-Pleiotropy residual sum and outlier methods for Coffee intake and diabetic complication

| **Exposure** | **Outcome** | **Egger_intercept** | **SE** | ***P* value** | **MR_PRESSO Global *P* value** |
| --- | --- | --- | --- | --- | --- |
| **Coffee intake** | Diabetic nephropathy | -0.01574849 | 0.01032107 | 0.137184 | 0.408 |
| **Coffee intake** | Glomerular filtration rate in diabetics | -0.0009343152 | 0.001707312 | 0.5885473 | 0.089 |
| **Coffee intake** | Type2 diabetes with renal complications | -0.01629158 | 0.01775522 | 0.365504 | 0.1933333 |
| **Coffee intake** | Type 1 diabetes with renal complications | 0.009934187 | 0.01980631 | 0.6192046 | 0.2766667 |
| **Coffee intake** | albumin-to-creatinine ratio | 0.001043831 | 0.01203703 | 0.9315126 | 0.3893333 |
